# An atypical weakly haemolytic strain of *Brachyspira hyodysenteriae* is avirulent and can be used to protect pigs from developing swine dysentery

**DOI:** 10.1186/s13567-019-0668-5

**Published:** 2019-06-19

**Authors:** Tom La, Nyree Dale Phillips, Flaminia Coiacetto, David John Hampson

**Affiliations:** School of Veterinary and Life Sciences, Murdoch University, Murdoch, WA 6150 Australia

## Abstract

The anaerobic intestinal spirochaete *Brachyspira hyodysenteriae* colonises the large intestine of pigs and causes swine dysentery (SD), a severe mucohaemorrhagic colitis. SD occurs worldwide, and control is hampered by a lack of vaccines and increasing antimicrobial resistance. *B. hyodysenteriae* strains typically produce strong beta-haemolysis on blood agar, and the haemolytic activity is thought to contribute to the pathogenesis of SD. Recently, weakly haemolytic variants of *B. hyodysenteriae* have been identified in Europe and Australia, and weakly haemolytic strain D28 from Belgium failed to cause disease when used experimentally to infect pigs. Moreover, pigs colonised with D28 and then challenged with virulent strongly haemolytic strain B204 showed a delay of 2–4 days in developing SD compared to pigs not exposed to D28. The current study aimed to determine whether Australian weakly haemolytic *B. hyodysenteriae* strain MU1, which is genetically distinct from D28, could cause disease and whether exposure to it protected pigs from subsequent challenge with strongly haemolytic virulent strains. Three experimental infection studies were undertaken in which no diseases occurred in 34 pigs inoculated with MU1, although mild superficial lesions were found in the colon in 2 pigs in one experiment. In two experiments, significantly fewer pigs exposed to MU1 and then challenged with strongly haemolytic virulent strains of *B. hyodysenteriae* developed SD compared to control pigs not previously exposed to MU1 (*p *= 0.009 and *p* = 0.0006). These data indicate that MU1 lacks virulence and has potential to be used to help protect pigs from SD.

**Electronic supplementary material:**

The online version of this article (10.1186/s13567-019-0668-5) contains supplementary material, which is available to authorized users.

## Introduction

Swine dysentery (SD) is a severe mucohaemorrhagic colitis of pigs that classically results from infection of the caecum and colon with the anaerobic strongly beta-haemolytic intestinal spirochaete *Brachyspira hyodysenteriae* [1]. In North America and Europe, SD also has occurred in pigs either naturally or experimentally infected with members of two other strongly haemolytic *Brachyspira* species, *Brachyspira hampsonii* and *Brachyspira suanatina* [2, 3]. Of the latter two, infections with *B. hampsonii* occur most commonly, particularly in North America [4], whilst infections with *B. suanatina* are rare and currently are only known to occur in Europe [5, 6]. On the other hand, infections with *B. hyodysenteriae* occur commonly throughout the world [7]. Uncontrolled SD can severely depress feed conversion efficiency in the grower/finisher phases, and cause mortalities. Besides production losses, the disease poses a potential animal welfare issue. Many contemporary strains of *B. hyodysenteriae* are resistant to previously effective antimicrobials, and as a result control is becoming increasingly more difficult [8]. Furthermore, no effective commercial vaccines are available.

The haemolytic activity of *B. hyodysenteriae* strains has been considered to be an important virulence factor [9–12]. Weakly haemolytic mutant strains of *B. hyodysenteriae* have been created: these had reduced virulence in pigs and offered partial protection from subsequent challenge with a virulent strain, with fewer pigs developing SD [13]. In recent years, naturally occurring weakly haemolytic strains of *B. hyodysenteriae* have been identified in pigs in Europe and Australia on farms that had no clinical disease, or only mild diarrhoeal disease of uncertain aetiology [14–16]. A weakly haemolytic strain (D28) from a herd in Belgium did not induce disease in experimentally infected pigs. Moreover, in pigs colonised with D28 and then challenged with virulent strongly haemolytic *B. hyodysenteriae* strain B204, the development of SD was delayed by an average of 2–4 days compared to pigs not previously exposed to the D28. Nevertheless, 28 of the 30 pigs in both groups succumbed to SD within 30 days, so there was not significant protection against disease [17].

The two main aims of the current study were to determine (i) whether a weakly haemolytic *B. hyodysenteriae* strain (MU1) isolated from a pig in Australia could colonise pigs without causing SD, and (ii) whether pigs exposed to MU1 would be protected following subsequent challenge with strongly haemolytic virulent *B. hyodysenteriae* strains. To address these aims, three studies were undertaken using experimentally infected pigs.

## Materials and methods

### Animals and housing

In each experiment, castrated male pigs (Large White × Landrace × Duroc) of approximately 8–10 kg body weight were purchased from a commercial piggery that was free of SD as determined by regular clinical observation and laboratory testing. On arrival at the Animal Isolation House at Murdoch University the pigs were weighed, ear-tagged, and faecal samples were taken and cultured to help exclude the possible presence of *B. hyodysenteriae* or other *Brachyspira* species. The pigs were randomly assigned to groups, with each group being housed in a single pen in different rooms of the isolation house. Strict biosecurity protocols, including the use of different sets of protective clothing and boots in the different rooms were maintained to prevent transmission of infection between the rooms. The pigs were fed ad libitum on a commercial grower diet containing wheat, lupins and meat meal (16% crude protein) that did not contain antimicrobials.

### Spirochaete strains

Weakly haemolytic *B. hyodysenteriae* strain MU1 was isolated from a pig in an Australian herd where clinical SD was not present [14]. The strain belongs to sequence type 151 (ST151) by multilocus sequence typing (MLST) [18, 19], and is of undetermined serogroup. Strongly haemolytic *B. hyodysenteriae* strains WA1, BW1, Vic2, NSW5, Q10 and NSW54 were isolated from Australian pigs in herds where clinical SD occurred. They belonged to ST36, S247, ST30, ST248, ST32, or unknown, respectively, and were of serogroups B, unknown, H, B, A, and unknown, respectively [20]. Strains from countries other than Australia were not used for experimental infections because Australian quarantine regulations only permit such experiments in high-level containment facilities.

### Experimental design

The overall experimental design is summarised in Table 1, with further details being described below.

**Table 1:** **Overview of the design of the three experiments**

Experiment number	No of groups (number of pigs per group)	Purpose	Experiment duration following exposure to MU1 (days)	Virulent challenge strains
1	2 (10)	Assess comparative virulence of MU1	49	NSW54
2	3 (12)	Assess virulence of MU1 AND Assess protection conferred by MU1	73	WA1, BW1, Vic2, NSW5, Q10 (“cocktail”)
3	2 (12)	Assess protection conferred by MU1 and duration	132 (for vaccinates), 117 for positive controls	WA1, BW1, Vic2, NSW5, Q10 (“cocktail”)

#### Experiment 1

This experiment was undertaken to determine the ability of MU1 to colonise pigs, and to assess its virulence. Twenty pigs were randomly assigned to two groups each of 10 pigs. Pigs in group A were inoculated with weakly haemolytic *B. hyodysenteriae* strain MU1 and group B were challenged with strongly haemolytic strain NSW54. Exposure to the spirochaetes occurred when the pigs had body weights of approximately 18–20 kg. The pigs were killed 49 days after the first day of the experimental exposure with the respective *B. hyodysenteriae* strains.

#### Experiment 2

This experiment was designed to confirm that colonisation with MU1 did not cause disease, to assess whether colonisation persisted for a prolonged period, and whether pigs inoculated with MU1 were protected from disease following challenge with virulent strains. Thirty-six pigs were randomly assigned to three groups each of 12 pigs. At an average body weight of approximately 10–12 kg the pigs in groups A and B were inoculated with strain MU1, whilst the pigs in group C were not inoculated. Thirty days after the inoculation with MU1, pigs in groups B and C were challenged with an equal mixture of the five strongly haemolytic *B. hyodysenteriae* strains WA1, BW1, Vic2, NSW5 and Q10 (a “cocktail”, not including strain NSW54 used in experiment 1). All remaining pigs were slaughtered 43 days after the first day of challenge with this cocktail.

#### Experiment 3

This experiment was designed to confirm whether pigs inoculated with MU1 were protected from disease following subsequent challenge with virulent strains. Twenty-four pigs were randomly assigned to two groups each of 12 animals. When the pigs reached an average body weight of 10–12 kg those in group A were inoculated with *B. hyodysenteriae* strain MU1, whilst the pigs in group B were not inoculated. Thirty-two days later the pigs in both groups were challenged with the “cocktail” of five strains used in experiment 2. Thirty-one days later all pigs in both groups were re-challenged with the same cocktail of strains. Pigs of group B that had not developed clinical signs were killed 85 days after the commencement of challenge with the cocktail. The pigs in group A were kept for a further 15 days (100 days after experimental infection) before being killed.

### Experimental infections

When the pigs reached the target weight for each experiment, they were challenged with *B. hyodysenteriae* strains by feeding each of them with 10 Trypticase Soy agar plates containing 5% (vol/vol) defibrinated ovine blood. The plates had been inoculated with the appropriate *B. hyodysenteriae* strains, incubated for 5 days and surface growth harvested from one plate into phosphate buffered saline to check for the presence of actively motile spirochaetal cells, and to undertake a cell count using a haemocytometer chamber which was observed under a phase contrast microscope. Each plate that was used came from a batch having an active log phase surface growth of ~10^8^ spirochaete cells. The agar from the plates was mixed into a handful of feed pellets, and each pig was challenged individually using a piglet feeding/drinking dish. The pigs were monitored until each had completely consumed the inoculum. This procedure was repeated daily on the following 4 days. Where a “cocktail” of five strains was used, the growth on 2 plates per strain was fed in the same manner (i.e. each pig received 10 plates in total).

### Health monitoring

Following experimental exposure to the spirochaetes, all pigs were visually monitored daily for signs of disease, and faeces was collected twice weekly for spirochaete culture and PCR. Samples were scores as 0 = normal; 1 = diarrhoea; 2 = diarrhoea with mucus; 3 = diarrhoea with mucus and fresh blood (dysentery). Once dysentery (score 3) was observed, the pigs were recorded as having SD and were removed for post-mortem examination (pm). The remaining pigs were killed at the times described for the individual experiments.

### Spirochaete detection

Faecal and colonic samples were plated onto selective Trypticase Soy Agar (BBL) plates containing 5% (vol/vol) defibrinated ovine blood, 400 μg/mL of spectinomycin, and 25 μg/mL each of colistin and vancomycin (Sigma-Aldrich) [21]. The plates were incubated for 5–7 days at 37 °C in an anaerobic environment of 94% H_2_ and 6% CO_2_ generated with anaerobic Gaspak plus sachets (BBL). The plates were examined for the presence of a low, flat, spreading growth and associated haemolysis. The extent of growth on the plates was scored on a scale from one to five, with one representing light growth in the inoculum and five indicating heavy growth through to the last streak on the isolation plate. Surface growth was re-suspending in phosphate-buffered saline and examined under a phase-contrast microscope. The harvested growth on plates suspected to have spirochaete growth were subjected to a PCR for *B. hyodysenteriae*, as previously described [22].

#### Identification of strains

The identity of the strains isolated from the colons of infected pigs in experiment 3 was determined using melting curves generated in a real-time PCR. To do this, the loci used in the *B. hyodysenteriae* MLST scheme [19] were examined to identify regions that could be used to discriminate between MU1 and the five strongly haemolytic challenge strains. Real-time PCR assays targeting the glycerol kinase gene (*glp*K) using the forward primer 5′-ATACAATGGGTAAGAGATGAAC-3′ and reverse primer 5′-CTCCATTAGTGTCTTTTACCTTTG-3′, and the thiolase gene (*thi*) using forward primer 5′-TGTGCATTTGAACGTTATC-3′ and reverse primer 5′-TTAAATCTTCCGCTTTTTACTG-3′ were designed and optimised for the RotorGene Q instrument (Qiagen). The RT-PCR was performed in two separate reactions. The *glpK* reaction was performed first to differentiated between WA1, BW1, NSW5, MU1 and Q10/Vic2. Q10 and Vic2 had the same g*lpK* sequence, so where Q10/Vic2 was recorded as positive the *thi* gene real time PCR was used to distinguish between them.

The QIAamp DNA Stool Mini Kit (Qiagen) was used to extract genomic DNA from *B. hyodysenteriae* strains grown from the colon contents of all infected pigs in experiment 3. The purified DNA (4 ng) samples were added to a reaction mixture consisting of 10 μL 2× Type-it HRM Master Mix (Qiagen), 720 nM of each primer, and ultrapure water to a final reaction volume of 20 μL. The PCR thermocycling parameters were as follows: initial denaturation at 95 °C for 5 min, 40 cycles with denaturation at 95 °C for 10 s and annealing at 50 °C for 30 s and extension at 72 °C for 10 s, followed by High Resolution Melt (HRM) ramping from 70 to 90 °C. Fluorescence data were acquired at 0.05 °C increments every 2 s in order to generate specific melting curves. MU1 and the five strongly haemolytic strains were included as melting curve standards. Reactions were performed in quadruplicate for reference strains. Data analysis was performed using the Rotor-Gene Q Software 2.1.

### Post-mortem examination

The pigs were stunned using a captive bolt pistol and exsanguinated by severing the carotid artery. The carcase was opened and the intestinal tract removed. The large intestine was opened along its length and intestinal contents were collected from the caecum and proximal colon for spirochaete culture. Observations of gross pathological changes in the caecum and colon and their distribution were recorded. The tissues were scored as [0], normal; [1], mild localised erythema; [2], mild superficial erosions; [3], moderate superficial erosions; [4], severe erosions with mucus and fresh blood. The extent and distribution of the lesions was recorded together with other observations such as the presence of fibrin associated with the lesions.

### Histological examination

In experiment 3, in addition to examining gross pathological changes, a tissue section from the proximal colon from each pig was collected and fixed in 10% (v/v) neutral buffered formalin for histological examination. The fixed sections were blocked, embedded in paraffin, cut at 4 µm and stained with haematoxylin and eosin.

The slides were examined by a specialist veterinary pathologist who was blinded to the origin of the sections. The entire length of each section was examined for the presence of mucosal erosion or ulceration. The total number of lymphoid follicles within the submucosa were counted for each section. Inflammatory changes within the mucosa were characterised by the type of inflammatory cell present, and were grouped according to the severity of the inflammatory cell infiltrate. Well-preserved sections of intestine with the mucosal glands (crypts) and enterocytes cut in longitudinal section were examined under 400× magnification. Measurements were made to quantify the number of intra-epithelial lymphocytes (granulated and non-granulated), the number of lymphocytes, plasma cells, granulocytes (eosinophils and neutrophils) and macrophages within the laminal propria, and severity of inflammation within the mucosa. An estimate of mucosal thickness was made by estimating the proportion of the field of view that the mucosa occupied at 100× magnification, and these were grouped according to whether the mucosa occupied 0–25%, 26–50%, 51–75%, 76–99% or 100% of the field of view. The presence of surface/intraglandular bacteria, amoebae (*Balantidium coli*) and any other infectious agents or abnormal histological changes were recorded.

### Analysis

For each experiment, Fisher’s exact test was used to make comparisons between pig groups in: (i) numbers of animals showing or not showing signs of disease; (ii) numbers that excreted spirochaetes post-exposure, (iii) numbers that were or were not culture positive at pm, and (iv) numbers that had or did not have gross large intestinal lesions at pm. Median values for daily scores of faecal excretion, clinical signs, and large intestinal lesions and colonisation at pm were calculated for the different groups in each experiment. The Mann–Whitney U test (two tailed) was used to compare the differences in the distribution of daily or pm values between pairs of groups in each experiment. In experiment 2 the results for group A initially were compared with those for group B. Subsequently, results for group B where compared with those for group C. These calculations were undertaken separately as each pair of comparisons focused on different questions being investigated in experiment 2.

## Results

### Experiment 1: MU1 colonises pigs but does not cause disease

#### Colonisation

None of the pigs were culture positive for *Brachyspira* species at the start of the experiment. Colonisation of the pigs after inoculation as indicated by faecal shedding of the respective strains is shown in Table 2. Of the pigs in group A that were inoculated with weakly haemolytic strain MU1, one pig was positive 10 days after the first day of inoculation and all were colonised by 24-days post-inoculation (pi). One pig stopped shedding *B. hyodysenteriae* after 28-days, three stopped shedding after 31 days and one after 42 days. The remaining five pigs were colonised for the duration of the experiment. The pigs had spirochaetes recovered from their faeces on 82 of the 150 samplings. For all pigs except pig 9, the number of spirochaetes in the faeces was less than in the pigs of positive control group B. All pigs in group B were colonised with NSW54 at 17-days post-challenge (pc), and these continued to be colonised for the duration of the experiment. Pigs had spirochaetes present in their faeces for 109 of the 150 samplings, and this was significantly more frequent than for the pigs in group A (*p* < 0.0001). On all sampling days from 35 to 45 days pi the colonisation of the pigs in group B challenged with NSW54 was significantly greater than in the pigs in group A challenged with MU1 (Mann–Whitney, *p *< 0.01). Comparisons of the median scores for the whole experimental period also indicated that colonisation was significantly greater in the pigs in group B than in those of group A (*p* < 0.01).

**Table 2:** **Shedding of** ***B. hyodysenteriae*** **in the faeces of pigs in experiment 1 as detected by selective anaerobic culture**

Pig group	Pig number	Culture results: days following first day of exposure^a^														
		0	3	7	10	14	17	21	24	28	31	35	38	42	45	Median^b^
A	1	0	0	0	0	1	1	1	2	2	2	2	1	1	1	1
	2	0	0	0	0	1	2	1	1	1	1	2	1	2	2	1
	3	0	0	0	0	0	0	1	1	2	1	0	0	0	0	0
	4	0	0	0	0	0	0	0	2	2	1	0	0	0	0	0
	5	0	0	0	0	1	1	2	2	2	1	1	0	1	1	1
	6	0	0	0	0	1	1	1	2	2	1	1	0	1	1	1
	7	0	0	0	0	0	0	1	1	1	1	1	1	1	0	0
	8	0	0	0	0	1	2	2	2	1	1	0	0	0	0	0
	9	0	0	0	1	1	1	5	5	3	3	3	3	2	2	2
	10	0	0	0	0	0	0	1	1	1	0	0	0	0	0	0
Median^c^		0	0	0	0	1	1	1	2	2	1	1**	0**	1**	1**	1**
B	11	0	0	0	1	4	4	4	4	4	4	4	4	4	4	4
	12	0	0	0	0	0	1	1	1	1	1	1	1	1	1	1
	13	0	0	0	0	1	1	2	1	1	1	1	1	1	1	1
	14	0	0	0	0	0	1	1	3	4	4	4	4	4	5	3
	15	0	0	0	0	0	1	1	1	1	1	1	1	1	1	1
	16	0	0	0	0	1	1	1	1	1	1	2	2	2	2	1
	17	0	0	0	0	3	3	5	4	4	4	4	4	4	4	4
	18	0	0	0	1	1	2	4	4	4	4	4	4	5	5	4
	19	0	0	0	0	0	1	1	1	2	2	3	3	3	4	1
	20	0	0	0	4	4	4	4	4	4	4	4	4	4	4	4
Median^c^		0	0	0	0	1	1	1.5	2	3	3	3.5**	3.5**	3.5**	4**	2**

Pigs in group A were inoculated with weakly haemolytic *B. hyodysenteriae* strain MU1 and those in group B were challenged with a strongly haemolytic field strain NSW54 isolated from a pig with SD.

^a^Culture score: 1 to 5, extent of growth on selective agar plates; 0, no growth.

^b^Median calculated using values from day 3–35 post challenge.

^c^Medians for the sampling days in groups A and B that are significantly different in a Mann–Whitney test are marked with asterisks (* *p* < 0.05; ** *p* < 0.01).

Colonisation of the caecum and colon at pm is shown in Table 3. The colons of all the pigs in both groups were colonised with the respective strains to which they had been exposed, but significantly more spirochaetes were found in the pigs infected with NSW54. The caecum of only one pig in the group inoculated with MU1 was colonised, compared to nine in the control group. The distribution of scores for colonisation in the caecum, colon and faeces were all significantly higher in the pigs challenged with NSW54 than in those exposed to MU1 (*p *< 0.01).

**Table 3:** **Clinical signs and gross lesions in the caecum and colon at pm 49** **days after exposure in the pigs in experiment 1**

Pig group	Pig number	Clinical signs^a^	Lesions observed at pm^b^		Culture^c^		
			Caecum	Colon	Caecum	Colon	Faeces
A	1	0	[0]	[0]	0	1	1
	2	0	[1] (patchy)	[2] (patchy, upper 1/3)	0	1	2
	3	0	[0]	[0]	0	1	0
	4	0	[0]	[0]	0	1	0
	5	0	[0]	[0]	0	1	1
	6	0	[0]	[0]	1	1	1
	7	0	[0]	[0]	0	1	0
	8	0	[0]	[0]	0	1	0
	9	0	[1] (patchy)	[2] (patchy, upper 1/3)	0	1	1
	10	0	[0]	[0]	0	1	0
Median^d^		0	0**	0	0**	1**	0**
B	11	1	[3]	[2] (localised, upper 1/3)	4	5	4
	12	0	[0]	[0]	1	3	1
	13	0	[0]	[0]	0	3	1
	14	1	[3]	[2] (localised, upper 1/3)	5	5	5
	15	0	[2]	[0]	3	3	1
	16	0	[2]	[0]	3	4	2
	17	1	[3]	[2] (localised, upper 1/3)	5	5	4
	18	1	[4]	[3] (upper 1/3)	5	5	5
	19	0	[3]	[0]	5	5	4
	20	1	[3]	[2] (localised, upper 1/3)	5	5	4
Median^d^		0.5	3**	1	4.5**	4.5**	4**

Pigs in group A were inoculated with weakly haemolytic *B. hyodysenteriae* strain MU1 and those in group B were challenged with a strongly haemolytic field strain NSW54 isolated from a pig with SD.

^a^Clinical signs: 0, normal; 1, diarrhoea; 2, diarrhoea with mucus; 3, dysentery.

^b^Lesion scores: [0] normal; [1] mild localised erythema; [2] mild superficial erosions; [3] moderate superficial erosions; [4] severe erosions with mucus and fresh blood.

^c^Culture score: 1 to 5, extent of growth on selective agar plates; 0, no growth.

^d^Medians for the values in groups A and B that are significantly different in a Mann–Whitney test are marked with asterisks (* *p* < 0.05; ** *p* < 0.01).

#### Disease

None of the 10 pigs in group A inoculated with MU1 showed signs of disease. Five pigs from the positive control group B challenged with NSW54 developed diarrhoea (score 1) during the 7-week post-inoculation period (Table 3). None developed dysentery (score 3) and so they were all killed at the designated end of the experiment. Differences between the groups in disease occurrence (diarrhoea) were statistically significant in Fisher’s exact test (*p* = 0.0325), but disease scores failed to reach a significantly difference in the Mann–Whitney test.

#### Gross lesions at pm

Observation of lesions in the colon and caecum of the pigs at pm are summarised in Table 3. Two pigs from group A had mild lesions in the caecum, compared to eight pigs from the positive control group B, including one having severe lesions. The same two pigs from group A had mild superficial lesions in the colon, compared to five pigs in the positive control group B that had mild to moderate gross lesions. The lesions in the colon seen in both groups were localised to the upper one-third (ascending colon). The caecums and colons of other pigs in both groups had a normal gross appearance. The distribution of scores for pathological changes in the caecum (but not the colon) were significantly greater in group B than in group A (*p *< 0.01).

### Experiment 2: MU1 persistently colonises pigs without causing disease, and provides significant protection against subsequent virulent challenge

#### Colonisation

None of the pigs were culture positive for *Brachyspira* species at the commencement of the experiment. For pigs in experiment 2, a summary of colonisation after inoculation as evidenced by faecal shedding is shown in Table 4. All the pigs in Group A except for pig 16 were identified as being colonised by MU1 at some point, and in each case growth on the plates was scored as one, indicating light growth. Excretion varied in duration, with one animal only being culture positive at one sampling time (pig 26) and another being culture positive with MU1 over a period of 40 days (pig 6). Four pigs were culture positive in the colon at pm (72 days pi with MU1), with slight to moderate growth of MU1 recorded (Table 5).

**Table 4:** **Shedding of** ***B. hyodysenteriae*** **in the faeces of pigs in experiment 2 as detected by culture on selective media**

Pig group	Pig number	Culture results: days after first day of inoculation with MU1^a^							Culture results: days after first day of challenge with five virulent strains^b^										Median (post cocktail)
		8	11	15	18	22	25	29	7	10	14	17	21	24	28	31	35	38	
A	1	0	0	0	1^d^	1^d^	0	0	0	0	0	0	0	0	0	0	0	0	0
	2	0	0	0	0	1^d^	1^d^	1^d^	1^d^	1^d^	1^d^	0	0	0	0	0	0	0	0
	3	0	0	1^d^	1^d^	1^d^	1^d^	1^d^	0	0	0	0	0	0	0	0	0	0	0
	4	0	0	0	0	1^d^	1^d^	0	0	0	0	0	0	0	0	0	0	0	0
	6	0	0	1^d^	1^d^	1^d^	1^d^	1^d^	1^d^	1^d^	1^d^	1^d^	1^d^	0	0	0	0	0	0.5
	7	0	0	0	1^d^	1^d^	1^d^	1^d^	0	0	0	0	0	0	0	0	0	0	0
	10	0	0	0	0	0	1^d^	1^d^	1^d^	0	0	0	0	0	0	0	0	0	0
	15	0	0	0	0	1^d^	0	1^d^	0	0	0	0	0	0	0	0	0	0	0
	16	0	0	0	0	0	0	0	0	0	0	0	0	0	0	0	0	0	0
	18	0	0	0	1^d^	1^d^	1^d^	0	0	0	0	0	0	0	0	0	0	0	0
	22	0	0	0	0	1^d^	1^d^	0	0	0	0	0	0	0	0	0	0	0	0
	26	0	0	0	0	1^d^	0	0	0	0	0	0	0	0	0	0	0	0	0
Median^c^		0	0	0	0	1	1	0.5	0	0	0	0	0	0	0	0	0	0	0
B	5	0	0	0	0	0	0	0	0	0	0	0	0	0	0	0	0	0	0
	9	0	0	0	1^d^	1^d^	1^d^	1^d^	1^d^	0	0	0	0	0	0	0	0	0	0
	12	0	0	0	0	0	1^d^	1^d^	0	0	0	0	0	0	0	5	5	Dead	0
	14	0	0	0	0	1^w^	0	0	0	0	0	0	0	0	0	0	4	5	0
	19	0	0	1^d^	1^d^	1^d^	1^d^	1^d^	1^d^	1^d^	1^d^	0	0	0	0	0	0	0	0
	23	0	0	0	1^d^	1^d^	1^d^	1^d^	1^d^	1^d^	1	1	1	0	0	0	0	0	0
	24	0	0	0	0	0	1^d^	0	1^d^	1^d^	1^d^	0	1	0	0	0	0	0	0
	25	0	0	0	1^d^	0	1^d^	1^d^	1^d^	1^d^	1^d^	0	0	0	0	0	0	0	0
	27	0	0	0	0	1^d^	1^d^	0	0	0	0	0	0	0	0	0	0	0	0
	29	0	0	1^d^	1^d^	1^d^	1^d^	1^d^	0	0	0	0	0	0	0	0	0	0	0
	32	0	0	0	0	1^d^	0	1^d^	1^d^	1^d^	1^d^	0	0	0	0	0	0	0	0
	33	0	0	1^d^	1^d^	1^d^	0	0	0	0	0	0	0	0	0	0	0	0	0
Median^c^		0	0	0	0.5^#^	1^##^	1^##^	1^#^	0.5	0	0	0	0	0^#^	0	0^#^	0^##^	0^#^	0^##^
C	8	0	0	0	0	0	0	0	0	0	0	0	0	5	5	5	5	Dead	0
	11	0	0	0	0	0	0	0	0	0	0	0	0	0	0	5	5	5	0
	13	0	0	0	0	0	0	0	0	0	0	0	0	0	5	5	5	5	0
	17	0	0	0	0	0	0	0	0	0	5	5	5	5	Dead	Dead	Dead	Dead	5
	20	0	0	0	0	0	0	0	0	3	5	5	5	5	Dead	Dead	Dead	Dead	5
	21	0	0	0	0	0	0	0	0	0	4	5	5	5	Dead	Dead	Dead	Dead	4.5
	28	0	0	0	0	0	0	0	0	0	0	0	0	5	5	5	5	Dead	0
	30	0	0	0	0	0	0	0	0	5	5	5	Dead	Dead	Dead	Dead	Dead	Dead	5
	31	0	0	0	0	0	0	0	0	0	0	0	0	5	5	5	5	Dead	0
	34	0	0	0	0	0	0	0	0	0	0	0	0	0	0	5	5	5	0
	35	0	0	0	0	0	0	0	0	0	0	0	0	0	0	0	5	5	0
	36	0	0	0	0	0	0	0	0	0	0	0	0	0	0	0	0	0	0
Median^c^		0	0	0	0^#^	0^##^	0^##^	0^#^	0	0	0	0	0	5^#^	2.5	5^#^	5^##^	5^#^	0^##^

Group A was only experimentally inoculated with weakly haemolytic *B. hyodysenteriae* strain MU1. Group B was inoculated with MU1 and subsequently challenged with a mixture of five virulent field strains of *B. hyodysenteriae*. Group C was only challenged with the virulent strains.

Dead—indicates pig that developed dysentery and was removed for pm.

^a^Culture score: 1 to 5, extent of growth on selective agar plates; 0, no growth.

^b^Group A exposed to MU1 and not challenged with the cocktail of strains.

^c^Medians for groups A and B that were significantly different in a Mann–Whitney test are marked with asterisks (* *p* < 0.05; ** *p* < 0.01). Medians for groups B and C that were significantly different in a Mann–Whitney test are marked with hashes (^#^*p* < 0.05; ^##^*p* < 0.01).

^d^Growth of weakly haemolytic *B. hyodysenteriae* (MU1). All others were strongly haemolytic.

**Table 5:** **Clinical signs and gross lesions observed at pm 43 days post-challenge with the cocktail in the caecum and colon of pigs in experiment 2**

Pig group	Pig number	Clinical signs^a^	Lesions observed at pm^b^		Culture^c^		
			Caecum	Colon	Caecum	Colon	Faeces
A	1	0	[0]	[0]	0	0	0
	2	0	[0]	[0]	0	0	0
	3	0	[0]	[0]	0	0	0
	4	0	[0]	[0]	0	0	0
	6	0	[0]	[0]	0	0	0
	7	0	[0]	[0]	0	0	0
	10	0	[0]	[0]	0	0	0
	15	0	[0]	[0]	0	3^e^	0
	16	0	[0]	[0]	0	1^e^	0
	18	0	[0]	[0]	0	1^e^	0
	22	0	[0]	[0]	0	3^e^	0
	26	0	[0]	[0]	0	0	0
Median^d^		0	0	0	0	0	0*
B	5	0	[0]	[0]	0	0	0
	9	0	[0]	[0]	0	5	3
	12	3	[3]	[4] (entire length)	0	5	5
	14	1	[0]	[3] (upper 1/3)	0	5	3
	19	0	[0]	[0]	0	5	5
	23	0	[0]	[2] (upper 1/3)	0	5	2
	24	0	[0]	[2] (upper 1/3)	0	5	3
	25	0	[0]	[0]	0	5^e^	5^e^
	27	0	[0]	[0]	0	0	0
	29	0	[0]	[0]	0	0	0
	32	0	[0]	[0]	0	0	0
	33	0	[0]	[0]	0	0	0
Median^d^		0^##^	0	0^##^	0^##^	5	2.5*^#^
C	8	3	[1]	[4] (plus fibrin, entire length)	4	5	5
	11	1	[0]	[3] (upper 1/3)	0	5	5
	13	1	[0]	[3] (upper 1/3)	2	5	5
	17	3	[4]	[4] (plus fibrin, entire length)	5	5	5
	20	3	[4]	[4] (plus fibrin, entire length)	5	5	5
	21	3	[4]	[4] (plus fibrin, entire length)	5	5	5
	28	3	[0]	[4] (plus fibrin, entire length)	3	5	5
	30	3	[4]	[4] (plus fibrin, entire length)	5	5	5
	31	3	[0]	[4] (plus fibrin, entire length)	5	5	5
	34	3	[1]	[4] (entire length)	5	5	5
	35	0	[0]	[0]	0	0	0
	36	0	[0]	[0]	0	5	1
Median^d^		3^##^	0.5	4^##^	4.5^##^	5	5^#^

Group A was experimentally inoculated with weakly haemolytic *B. hyodysenteriae* strain MU1. Group B was inoculated with MU1 and subsequently challenged with a mixture of five virulent field strains of *B. hyodysenteriae*. Group C was only challenged with the virulent strains.

^a^Clinical signs: 0, normal; 1, diarrhoea; 2, diarrhoea with mucus; 3, dysentery.

^b^Lesion scores: [0] normal; [1] mild localised erythema; [2] mild superficial erosions; [3] moderate superficial erosions; [4] severe erosions with mucus and fresh blood.

^c^Culture score: 1 to 5, extent of growth on selective agar plates; 0, no growth.

^d^Medians for groups A and B that were significantly different in a Mann–Whitney test are marked with an asterisk (* *p* = 0.05). Medians for groups B and C that were significantly different in a Mann–Whitney test are marked with hashes (^#^*p* < 0.05; ^##^*p* < 0.01).

^e^Growth of weakly haemolytic *B. hyodysenteriae* (MU1). All others were strongly haemolytic.

For group B, all but one pig (pig 5) shed MU1 prior to the subsequent challenge with the cocktail of virulent strains. Four of these pigs (19, 24, 25 and 32) shed low numbers of weakly haemolytic MU1 for up to 14 days after challenge with the virulent strains. Subsequently, 31 days pc with the cocktail of virulent strains, pig 12 excreted large numbers of strongly haemolytic spirochaetes, and by 35-days pc pigs 12 and 14 were excreting large numbers. Pigs 23 and 24 excreted low numbers of strongly haemolytic spirochaetes for between 1 and 3 sampling points, before becoming negative. Seven pigs in group B had moderate to high numbers of spirochaetes in their faeces at pm (Table 5), and this was the only time that groups A and B showed significant differences in faecal excretion (Mann–Whitney: *p *< 0.05). These seven pigs, including three without any gross lesions in the colon, also had large numbers of spirochaetes recovered from their colons. In all but pig 25 these isolated spirochaetes were strongly haemolytic. Spirochaetes were not recovered from the caecum of any pig.

Of the 12 pigs in group C that were just challenged with the cocktail of virulent strains, 11 showed high rates of excretion with large numbers of spirochaetes over the experimental period. The earliest that excretion was recorded was 10-days pc. Overall 10 of the 12 pigs had heavy growth of the spirochaete in their faeces at pm, and this excretion rate was significantly higher than in the pigs of group B (*p *< 0.05). Eleven of the 12 pigs had heavy spirochaetal growth in the colon at pm, and nine had moderate to heavy growth in the caecum. In both cases these values were significantly greater than for the pigs of group B (Table 5).

#### Disease

None of the pigs from group A developed diarrhoea or dysentery (Table 5). One pig in Group B developed diarrhoea, and one developed dysentery and so was removed. In Group C, two pigs developed diarrhoea and eight developed dysentery. The number of pigs with SD in group C (8 of 12) was statistically significantly greater than the number of pigs of group B that previously had been inoculated with MU1 (1 of 12) (*p *= 0.009) (Fisher’s exact test). If diarrhoea was included, rates of disease again were significantly higher in pigs from group C (10 of 12) than in pigs from group B (2 of 12) (*p *= 0.003). These differences between groups B and C were also significantly different in the Mann–Whitney test (*p* < 0.01), indicating that prior exposure to MU1 was protective against challenge with virulent strains of *B. hyodysenteriae*.

#### Gross lesions at pm

None of the pigs in group A showed lesions in the caecum or colon at pm (Table 5). In group B, the pig with dysentery had severe mucohaemorrhagic lesions in the caecum and colon at pm, and the pig with diarrhoea had moderate lesions. Two other pigs had mild localised lesions in the upper colon. Eight of the pigs in group C had severe mucohaemorrhagic colitis, two had more moderate lesions, and two had no lesions. Comparison of frequency of moderate and severe lesions in the groups found no significant differences in outcome between groups A and B (*p* = 0.478), but there were significant differences between groups B and C (*p *= 0.003). The significance of the differences in occurrence of lesions in groups B and C was confirmed for the colon but not the caecum using the Mann–Whitney test (*p *< 0.01).

### Experiment 3: MU1 again provides significant protection against SD

#### Colonisation

None of the pigs were culture positive for *Brachyspira* species at the commencement of the experiment. A summary of the faecal excretion of pigs in Group A over a 32-day period pi with MU1 is shown in Table 6. Nine of the 12 pigs (75%) were colonised, in each case with growth on the plates scored as one, indicating light growth. Faecal excretion varied in duration, with animals being culture positive for between 3 and 6 sampling times over the period. As anticipated, none of the unchallenged pigs in group B shed spirochaetes over this 32-day period.

**Table 6:** **Shedding of weakly haemolytic** ***B. hyodysenteriae*** **MU1 in the faeces of pigs in group A in experiment 3, as detected by culture on selective medium**

Pig number	Culture results (days after first day of challenge with MU1)^a^								Median
	8	11	15	18	22	25	29	32	
1	0	0	0	0	0	0	0	0	0
2	0	0	1	1	1	1	1	1	1
3	0	0	0	1	1	1	1	1	1
4	0	0	0	0	1	1	1	1	0.5
5	0	0	0	1	1	1	1	1	1
6	0	0	0	0	0	0	0	0	0
7	0	0	0	0	0	1	1	1	0
8	0	0	0	1	1	1	0	1	0.5
9	0	0	0	0	1	1	1	0	0
10	0	0	0	0	0	0	0	0	0
11	0	0	0	1	1	1	0	1	0.5
12	0	0	0	0	1	1	1	1	0.5
Median	0	0	0	0	1	1	1	1	0.5

Pigs subsequently were challenged with a cocktail of 5 virulent strains.

^a^Culture score: 1 to 5, extent of growth on selective agar plates; 0, no growth.

After the initial 32 days, the pigs in both groups were challenged with the cocktail of virulent strains. As no disease occurred, this challenge was repeated after another 31 days. Eight of the pigs originally inoculated with MU1 (group A) excreted or continued to excrete the weakly haemolytic strain in their faeces for varying periods, but for up to 48 days in pig 2 (Table 7). Only three pigs in group A (pigs 7, 9 and 10) and two of the pigs in group B (pigs 19 and 21) did not excreted spirochaetes over the period. Two other pigs in group B only started excreting following the second round of challenge (pigs 16 and 23), whereas the others that shed were doing this before the second challenge, despite disease not having occurred. The 10 colonised pigs in group B remained colonised for prolonged periods of over 50 days in some cases, until dysentery occurred and they were removed. The results of the Mann–Whitney tests confirmed that the distribution of faecal excretion was significantly greater in group B than in group A on each sampling point from 31 to 81 days pc (Table 7).

**Table 7:** **Faecal shedding of** ***B. hyodysenteriae*** **by pigs in experiment 3 during the challenge period, as detected by culture on selective media**

Group number	Pig number	Culture results (days after first day of first challenge with the virulent strains)^a^																											Median
		7	10	14	17	21	24	28	31	*35*	*38*	*41*	*45*	*48*	*51*	*55*	*58*	*61*	*65*	*68*	*71*	*75*	*78*	*81*	*85*	*88*	*91*	*95*	
A	1	0	0	0	0	0	0	0	0	0	0	0	0	0	0	0	0	0	0	0	0	0	0	0	0	0	1	3	0
	2	1^c^	1^c^	1^c^	1^c^	1^c^	1^c^	1^c^	1^c^	1^c^	1^c^	1^c^	1^c^	1^c^	0	0	0	0	0	0	0	0	0	0	0	0	0	0	0
	3	1^c^	1^c^	1^c^	0	0	0	0	0	0	0	0	0	0	0	0	0	0	0	0	0	0	0	0	0	0	0	0	0
	4	1^c^	1^c^	1^c^	1^c^	0	1^c^	1^c^	1^c^	1^c^	1^c^	1^c^	1^c^	0	0	0	0	0	0	0	0	0	0	0	0	0	0	0	0
	5	1	1^c^	1^c^	0	1^c^	1^c^	0	0	0	0	0	0	0	0	0	0	0	0	0	0	0	0	0	0	0	0	0	0
	6	0	1^c^	1^c^	1^c^	0	0	1^c^	0	0	0	0	0	0	0	0	0	0	0	0	0	0	0	0	0	0	0	0	0
	7	0	0	0	0	0	0	0	0	0	0	0	0	0	0	0	0	0	0	0	0	0	0	0	0	0	0	0	0
	8	1^c^	1^c^	1^c^	1^c^	1^c^	0	0	0	0	0	0	0	0	0	0	0	0	0	0	0	0	0	0	0	0	0	0	0
	9	0	0	0	0	0	0	0	0	0	0	0	0	0	0	0	0	0	0	0	0	0	0	0	0	0	0	0	0
	10	0	0	0	0	0	0	0	0	0	0	0	0	0	0	0	0	0	0	0	0	0	0	0	0	0	0	0	0
	11	1^c^	1^c^	1^c^	0	1^c^	0	0	0	1^c^	0	0	0	0	0	0	0	0	0	0	0	0	0	0	0	0	0	0	0
	12	1^c^	0	1^c^	1^c^	1^c^	1^c^	0	0	0	0	0	0	0	0	0	0	0	0	0	0	0	0	0	0	0	0	0	0
Median^b^		1*	1*	1	0	0	0	0	0*	0*	0*	0*	0**	0**	0**	0**	0**	0**	0**	0**	0**	0**	0**	0**	0	0	0	0	0**
B	13	0	0	0	0	0	0	0	2	2	2	2	2	2	2	2	2	2	3	5	5	5	5	5	Dead				2
	14	0	0	3	4	4	4	4	3	3	4	5	5	5	5	5	5	5	5	Dead									4
	15	0	0	0	0	0	0	0	2	2	2	2	3	3	4	4	5	5	5	5	5	5	5	5	Dead				3
	16	0	0	0	0	0	0	0	0	0	0	0	3	3	3	2	4	5	5	5	5	5	5	5	Dead				2
	17	0	0	0	0	0	2	2	2	2	2	2	2	2	2	3	1	1	1	5	5	5	5	5	dead				2
	18	0	0	0	0	3	3	3	3	3	4	5	5	5	5	5	5	5	5	5	5	Dead							4.5
	19	0	0	0	0	0	0	0	0	0	0	0	0	0	0	0	0	0	0	0	0	0	0	0	Dead*				0
	20	0	0	0	0	0	1	2	2	2	2	1	1	1	0	0	0	0	0	0	0	1	1	1	Dead				0
	21	0	0	0	0	0	0	0	0	0	0	0	0	0	0	0	0	0	0	0	0	0	0	0	Dead*				0
	22	0	0	0	0	0	0	2	2	2	1	1	2	1	1	2	2	1	1	1	3	3	4	5	Dead				1
	23	0	0	0	0	0	0	0	0	0	0	0	4	5	5	5	5	5	5	5	5	Dead							0
	24	0	0	3	3	3	3	3	3	4	4	4	5	5	5	5	5	5	5	Dead									4
Median^b^		0*	0*	0	0	0	0	1	2*	2*	2*	1.5*	2.5**	2.5**	2.5**	2.5**	3**	3.5**	4**	5**	5**	4**	4.5**	5**					2**

Pigs in group A had been exposed to MU1, and subsequently they and the pigs in group B which had not been exposed to MU1 were challenged with a cocktail of five virulent field strains of *B. hyodysenteriae*. Both groups were challenged with the cocktail again after 32 days. Surviving pigs in group A were killed 100 days following challenge with the cocktail, whilst those in group B were killed 85 days pc.

Dead—indicates pigs developed dysentery and were removed for pm.

Dead*—indicates 2 pigs unexposed to MU1 that were healthy and were killed after 85 days (end of experiment for group B).

^a^Culture score: 1 to 5, extent of growth on selective agar plates; 0, no growth. Days marked in italics are after the second inoculation with the cocktail, which occurred 32 days after the first inoculation with the cocktail.

^b^Medians for the values in groups A and B that are significantly different in a Mann–Whitney test are marked with asterisks (* *p* < 0.05; ** *p* < 0.01).

^c^Growth of weakly haemolytic *B. hyodysenteriae* (MU1). All others were strongly haemolytic.

At pm, 132-days pi with MU1, four pigs in group A had this strain identified in their colons (Table 8). The strongly haemolytic strains isolated from the colons of three other pigs in group A were BW1 (2 pigs) or WA1. Pig 1 developed dysentery and had moderate numbers of BW1 in the colon, whilst all the other pigs in group A had low numbers of spirochaetes present. Pig 1 was the only one that had *B. hyodysenteriae* recovered from its faeces, and none had *B. hyodysenteriae* recovered from their caecum. In comparison, all but two pigs in group B had *B. hyodysenteriae* in their colons, and these two did not develop disease. Nine pigs had heavy spirochaetal growth in the colon, and one had light growth. Four pigs also had *B. hyodysenteriae* recovered from their caecum. For the 10 pigs with spirochaetal growth in the colon, different strains were isolated from different pigs. Vic2 and BW1 each were isolated from three pigs, and WA1 and NSW5 each were isolated from two pigs. Q10 was not identified as the strain colonising any of the pigs. Spirochaete scores were significantly higher in the faeces and colon of pigs in group B than those in group A (*p *= 0.01), but this difference was not significant in the caecum.

**Table 8:** **Clinical signs and gross lesions observed at pm in the caecum and colons of pigs from experiment 3**

Group number	Pig number	Clinical signs^a^	Lesions observed at pm^b^		Culture^c^			Haemolysis (colon)	Identified strain
			Caecum	Colon	Caecum	Colon	Faeces		
A	1	1	[0]	[3]	0	3	3	Strong	BW1
	2	0	[0]	[0]	0	2	0	Weak	MU1
	3	0	[0]	[0]	0	0	0	–	–
	4	0	[0]	[0]	0	1	0	Weak	MU1
	5	0	[0]	[0]	0	1	0	Weak	MU1
	6	0	[0]	[1]	0	1	0	Strong	WA1
	7	0	[0]	[0]	0	0	0	–	–
	8	0	[0]	[0]	0	0	0	–	–
	9	0	[0]	[0]	0	0	0	–	–
	10	0	[0]	[2]	0	2	0	Strong	BW1
	11	0	[0]	[0]	0	1	0	Weak	MU1
	12	0	[0]	[0]	0	0	0	–	–
Median^d^		0**	0	0**	0	1**	0**		
B	13	3	[0]	[2]	0	5	5	Strong	Vic 2
	14	3	[1]	[4]	3	5	5	Strong	BW1
	15	3	[1]	[3]	1	5	5	Strong	WA1
	16	3	[0]	[3]	0	5	5	Strong	Vic 2
	17	3	[0]	[3]	0	5	5	Strong	BW1
	18	3	[1]	[4]	1	5	5	Strong	NSW 5
	19	0	[0]	[0]	0	0	0	–	–
	20	3	[0]	[3]	0	1	5	Strong	BW1
	21	0	[0]	[0]	0	0	0	–	–
	22	1	[0]	[3]	0	5	5	Strong	WA1
	23	3	[1]	[4]	0	5	5	Strong	Vic 2
	24	3	[1]	[4]	4	5	5	Strong	NSW 5
Median^d^		3**	0	3**	0	5**	5**		

Group A was exposed to MU1 and subsequently challenged with a mixture of five virulent field strains of *B. hyodysenteriae*. Group B was only challenged with the virulent isolates.

^a^Clinical signs: 0, normal; 1, diarrhoea; 2, diarrhoea with mucus; 3, dysentery.

^b^Lesion scores: [0] normal; [1] mild localised erythema; [2] mild superficial erosions; [3] moderate superficial erosions; [4] severe erosions with mucus and fresh blood.

^c^Culture score: 1 to 5, extent of growth on selective agar plates; 0, no growth.

^d^Medians for the values in groups A and B that are significantly different in a Mann–Whitney test are marked with asterisks (* *p* = 0.05; ** *p* = 0.01).

#### Disease

None of the pigs in group A that were inoculated with MU1 developed disease prior to challenge with the cocktail, and only one developed disease after this challenge. In the last few days of the experiment, 91-days pc with the cocktail, pig 1 which was one of three that had not excreted MU1 developed diarrhoea at the same time as it started to shed *B. hyodysenteriae*. The other pigs in group A remained healthy for the duration of the experiment (100 days pc with the cocktail). In group B, nine of the pigs developed dysentery (score 3), and one had diarrhoea when it was killed at 85-days pc. The other two pigs did not show clinical signs. The differences in occurrence of disease between groups A and B following their challenge with the cocktail was highly significant in Fisher’s exact test (*p* = 0.0006), and in the Mann–Whitney test (*p *< 0.01).

#### Gross lesions at pm

Pig 1, the animal with diarrhoea in group A, had moderate mucohaemorrhagic lesions in the colon at pm (Table 8). The other two pigs that had not excreted MU1 did not show clinical signs of SD, but at pm they had mild localised lesions in the upper colon (pig 10) or mild patchy erythema through the colon (pig 6). *B. hyodysenteriae* isolates cultured from the colon of these three pigs (1, 6 and 10) were strongly haemolytic. None of the other pigs in group A had gross pathological changes.

At pm, four of the pigs in group B had severe mucohaemorrhagic colitis, with moderate lesions found in five other pigs. One pig had mild localised lesions and two pigs had no lesions. The latter two did not have *B. hyodysenteriae* detected in their colons and they did not excrete *B. hyodysenteriae* in the faeces after experimental challenge. Overall, a highly significant difference occurred between groups A and B in terms of pathological changes, with 1 out of 12 pigs in group A and 9 out of 12 pigs in group B having moderate or severe erosive lesions at pm (*p* = 0.0028). Differences between the two groups were also significant in the Mann–Whitney test (*p* = < 0.01) for the colon, but not the caecum.

#### Histopathology

Histological findings for the pigs in experiment 3 are recorded in Additional files 1 and 2. Of the three pigs in group A that had gross changes observed in the colon, pig 1 that had moderate changes recorded had a moderate thickening of the mucosa, a slight increase in crypt numbers, no erosions or ulcerations and only rare surface mucosal epithelial cell injury. Of the other two, pig 6 had mild epithelial cell injury, and pig 4 had a mild increase in lymphocytes and plasma cells. None of the other 9 pigs had pathological changes or changes in inflammatory cell numbers. In the pigs of group B, of the 10 with gross lesions all but pig 6 had thickening of the mucosa. The two pigs without gross lesions also did not have mucosal thickening. Numbers of crypts were greater in nearly all pigs in group B than in the pigs from group A. Epithelial erosions, mild to moderate inflammation, and surface epithelial cell injury was observed in all 10 pigs of group B with gross changes. One of the two pigs in the group that did not have gross lesions had mild inflammation recorded. Most of the pigs in group B had mild to moderate increases in numbers of lymphocytes and plasma cells, macrophages and neutrophils. All but two of the pigs in group A had a few bacterial cells close to the epithelial surface, whilst two showed a surface “lawn” of bacteria (neither pig had colonic lesions). In group B, 6 pigs had a surface lawn of bacteria and six only had a few cells present close to the epithelium. The pigs with mild to moderate epithelial lesions were more likely to have a surface lawn of bacteria present.

## Discussion

This study had two main aims, the first of which was to determine whether or not weakly haemolytic *B. hyodysenteriae* strain MU1 from Australia could colonise and cause disease in pigs. Naturally avirulent or weakly virulent strains of *B. hyodysenteriae* have been known to exist for many years [23–26], and mutants that have been attenuated in different ways to reduce their virulence have been engineered [13, 27]. Haemolysin(s) produced by *B. hyodysenteriae* appear to be an important virulence factor, and natural weakly haemolytic strains recently have been identified in pig herds where there is little or no disease [14–16]. One weakly haemolytic strain from Belgium (D28) has been shown to be avirulent in experimentally infected pigs [17], and consequently it was hypothesised that Australian strain MU1 also may be avirulent, even if it belonged to a different genetic subgroup to D28 (MU1 is ST151 and D28 is ST172). D28 is a member of a clade of weakly haemolytic *B. hyodysenteriae* isolates that to date has only been described in Europe [28].

Most pigs (40/46: 87%) in all three experiments became colonised with MU1 following experimental inoculation, as evidenced by faecal excretion. MU1 numbers in the faeces and large intestine of colonised pigs generally were low, whereas pigs challenged with strongly haemolytic strains typically were much more heavily colonised, particularly a few days prior to and during the time the pigs developed disease. In a direct comparison of pigs of the same age exposed at the same time in experiment 1, MU1 also was excreted on significantly fewer days than was NSW54. This apparently less heavy colonisation with MU1 compared to a virulent strain was consistent with the results previously obtained for weakly haemolytic strain D28 [17]. In four pigs in experiment 2, MU1 was still found in the colon 132-days pi, even though it was not detected in the faeces. This is important as faecal screening for this potential “vaccine” strain likely lacks sensitivity for detection of all colonised animals.

Importantly, none of the 46 pigs inoculated with MU1 in the three experiments developed disease (before challenge with virulent strains in the case group B in experiment 2), although mild superficial lesions (score 2) were found in the colon in two colonised pigs in experiment 1. Mahu et al. [17] also found some slight hyperaemia in the colons of some pigs colonised by D28. The histological findings in our experiment 3 were consistent with the observations on gross pathology made at pm, confirming a lack of pathological changes in pigs exposed to MU1. The current results strongly suggest that MU1, like D28, has little capacity to cause disease. In turn this adds weight to the likelihood that production of strong haemolysis is an important virulence determinant in *B. hyodysenteriae*, although further work is required to investigate the mechanisms involved.

The second main aim of the study was to determine whether MU1 had potential as a live “vaccine” strain. Since the discovery of *B. hyodysenteriae* in the early 1970s there have been numerous attempts to produce vaccines to prevent pigs from developing SD. These include inactivated bacterins [29–31], which sometimes have been pepsin digested [32], recombinant surface proteins [33, 34], DNA vaccines [35], and the use of live avirulent strains given orally, sometimes boosted with bacterins [17, 36, 37]. Despite occasional reported successes with these vaccines in mice or in pig pen trials, and the great economic importance of SD, to date no commercial vaccines that have been developed have remained on sale for long. This emphasises the difficulty in producing efficacious vaccines for use in the field.

In experiments 2 and 3, where a total of 24 pigs were inoculated with MU1 and subsequently challenged with a cocktail of five strongly haemolytic virulent strains of *B. hyodysenteriae*, only three pigs (12.5%) developed disease (one with dysentery and one with diarrhoea in experiment 2; one with diarrhoea in experiment 3). On the other hand, of the 24 positive control pigs in these two experiments that were challenged with the cocktail of strains without being previously inoculated with MU1, 20 (83.3%) developed disease (eight with dysentery and two with diarrhoea in experiment 2; nine with dysentery and one with diarrhoea in experiment 3). In both experiments these differences in disease occurrence were highly significant (*p *= 0.012 and *p *= 0.0006, respectively). Furthermore, the only pig exposed to MU1 in experiment 3 that developed diarrhoea (pig 1) only showed clinical signs 95 days after first challenge with the virulent strains. Taken together, these experiments demonstrate that exposure to and colonisation with MU1 can offer a significant level of protection that may last for up to 3 months under the conditions of these experiments. In contrast, in the previous experiment where live weakly haemolytic D28 was used to “vaccinate” pigs, 28 of 30 vaccinated pigs developed disease within 30-days pc with virulent strain B204 [17]. On average the onset of disease in the vaccinated pigs in that study was delayed for approximately 2–4 days compared to unvaccinated pigs, with the duration depending on the challenge protocol. It is important to note that highly virulent strain B204 was used as the challenge, and it was delivered either by oral inoculation or by a “seeder” approach whereby pigs infected with B204 were mixed with the experimental pigs to infect them. This robust challenge methodology also resulted in 28 of the 30 unvaccinated pigs developing SD within 30 days. It would be informative to test MU1 under the same conditions used in Belgium, and also to examine the efficacy of D28 under the experimental conditions we describe here. The milder challenge that we used is not necessarily invalid, as it did eventually induce SD in 83.3% of the pigs not previously exposed to MU1. The relatively slow onset of disease and its inconsistent occurrence in the challenged pigs in this study may reflect the challenge strains used, the way the inoculum was prepared (agar plates compared to broth culture), or other pig-related or diet-related factors. Importantly, in natural outbreaks of SD on farms it is uncommon for a large proportion of animals to rapidly become diseased, and in this sense the outcomes mimicked the development of natural disease.

In experiments 2 and 3, five different strains of *B. hyodysenteriae* were used to challenge the pigs. This was done in order to help determine whether MU1 conferred protection against different strains belonging to different genetic backgrounds and serogroups. Strain NSW54 was not used as part of the cocktail as it had only caused mild disease and lesions in a few pigs in experiment 1, and so was not likely to be a highly virulent strain. In experiment 3, the strains that were isolated from the colons were identified by melt curve analysis. PCRs based on the *nox* gene were not used to differentiate between the *B. hyodysenteriae* strains because the *nox* gene is too highly conserved within the species to allow this differentiation. Using the melt curve analysis, different pigs had one or other of four different strains identified. This finding helps to confirm the broad protection conferred by MU1, and that the different challenge strains were able to cause disease. It is important to note that the methodology only identified the isolated strain, and the possibility that low numbers of other strains may have been present in the colon cannot be excluded. It has previously been shown that PCR by itself is not a sensitive method of detecting all of the *Brachyspira* spp. that are present in a given sample [38]. It is uncertain why different virulent strains came to predominated in different infected pigs, and improving understanding about the dynamics of these strain interactions is an important area for future investigation.

The mechanism(s) of the protection conferred by MU1 are still not entirely clear. The Belgian study using strain D28 suggested that it induced mucosal immunity, as pigs colonised with D28 (11 or 30 pigs) showed increases in faecal IgA in ELISAs that used D28 and B204 whole cells as antigen; furthermore, higher IgA levels were associated with a delay in development of SD [17]. Potentially if this mucosal immunity could be further stimulated it might help to prevent colonisation and disease. Mucosal antibody was not evaluated in the current study, but some observations were made that suggest that MU1 may have competed with and excluding the pathogenic strains. The existence of competition between strains was shown in experiment 3, as different pigs had different dominant *B. hyodysenteriae* strains in their colons—including strain MU1.

Some temporal evidence for competitive exclusion came from experiment 2, where only four of 12 pigs in group A that were exposed to the MU1 still had low to moderate numbers of this strain in their colons after 73 days. Importantly, this was the time that disease was occurring in the pigs in group B (that had been challenged with virulent strains), and this possibly could have been associated with a reduction in the presence of MU1 in these animals too. Another interesting observation occurred with pig 23 in group B, which excreted low numbers of MU1 until 14-days pc with the cocktail, and then excreted low numbers of strongly haemolytic *B. hyodysenteriae* at the next three sampling days before becoming negative. A closer observation of the dynamics of these strains in the large intestine of this pig at this time could provide important insights into the mechanisms of protection conferred by MU1. In experiment 3, the first challenge with the cocktail of strains did not provide protection against a subsequent challenge, and this argues against an immune mediated form of protection. On the other hand, MU1 was still found in the colon of four of the 12 pigs in group A when they were killed 132 days after they first had been exposed to this strain. At that time five other pigs were not colonised, and the remaining three had a pathogenic strain present. As in experiment 2, the fact that the four pigs colonised with MU1 did not develop SD suggests that the vaccine strain may have successfully competed with the challenge strain in the large intestine. The five pigs that were not colonised at pm probably were colonised earlier, at the time of challenge with the virulent strains, and this may have protected these pigs.

Another important observation from this work is that colonisation with MU1 was prolonged in some pigs, but not all. It is possible that increased numbers of pigs could be protected for longer periods by boosting with additional doses of MU1 later during the pig growth cycle. Boosting the MU1 vaccination using bacterins, recombinant proteins or DNA vaccines also might broaden and extend the window of immunological protection, although this could be detrimental if it interfered with colonisation by MU1. Finally, further work is required to evaluate different delivery systems, dose rates and other important parameters that need to be understood for developing MU1 into a successful live strain that protects against SD.

## Additional files

**Additional file 1.** **Histopathological changes observed at pm in the large intestine of pigs from experiment 3**. Pigs marked in bold had gross pathological changes observed at pm.

**Additional file 2.** **Changes in cell numbers observed at pm in the large intestine of pigs in experiment 3.** Pigs marked in bold had gross pathological changes observed at pm.

## Abbreviations

hpf: high powered field
MLST: multilocus sequence typing
pc: post-challenge
pi: post-inoculation
pm: post-mortem examination
SD: swine dysentery
