# Supplementary material for: An atypical weakly haemolytic strain of Brachyspira hyodysenteriae is avirulent and can be used to protect pigs from developing swine dysentery
Source: Vet Res. 2019 Jun 19;50:47. doi: 10.1186/s13567-019-0668-5 (PMC6585146; doi:10.1186/s13567-019-0668-5)
Supplement: Supplementary file 1 — Additional file 1. Histopathological changes observed at pm in the large intestine of pigs from experiment 3. Pigs marked in bold had gross pathological changes observed at pm. [file 13567_2019_668_MOESM1_ESM.docx]

**Additional file 1 Table showing histopathological changes observed at pm in the large intestine of pigs from experiment 3**. Pigs marked in bold had gross pathological changes observed at pm.

| Pig group | Pig number | Section length  (mm) | Submucosal lymphoid follicles per section | Mucosal thickness^a^ | Number of crypts^b^ | Erosion^c^ | Ulceration^c^ | Inflammation^d^ | Surface mucosal epithelial cell injury^d^ | Glandular bacterial cells |
| --- | --- | --- | --- | --- | --- | --- | --- | --- | --- | --- |
| A | **1** | **8** | **2** | **26-50** | **8** | **N** | **N** | **WNL** | **Rare** | **Few close to surface** |
|  | 2 | 7 | 0 | 0-25 | 6 | N | N | WNL | WNL | Few close to surface |
|  | 3 | 13 | 0 | 0-25 | 6 | N | N | WNL | WNL | Surface "lawn" and few close to surface |
|  | 4 | 17 | 2 | 0-25 | 6 | N | N | Mild | WNL | Few close to surface |
|  | 5 | 11 | 1 | 0-25 | 7 | N | N | WNL | WNL | Few close to surface |
|  | **6** | **12** | **0** | **0-25** | **7** | **N** | **N** | **WNL** | **Mild** | **Few close to surface** |
|  | 7 | 11 | 0 | 0-25 | 6 | N | N | WNL | WNL | Few close to surface |
|  | 8 | 13 | 0 | 0-25 | 7 | N | N | WNL | WNL | Few close to surface |
|  | 9 | 16 | 0 | 0-25 | 5 | N | N | WNL | WNL | Surface "lawn" and few close to surface |
|  | **10** | **14** | **1** | **0-25** | **6** | **N** | **N** | **WNL** | **WNL** | **Few close to surface** |
|  | 11 | 13 | 0 | 0-25 | 7 | N | N | WNL | WNL | Few close to surface |
|  | 12 | 14 | 0 | 0-25 | 6 | N | N | WNL | WNL | Few close to surface |
| B | **13** | **15** | **0** | **0-25** | **7** | **N** | **N** | **Mild** | **Rare** | **Few close to surface** |
|  | **14** | **18** | **0** | **51-75** | **11** | **Y** | **N** | **Moderate** | **Mild** | **Few close to surface** |
|  | **15** | **21** | **0** | **51-75** | **8** | **Y** | **N** | **Moderate** | **Mild** | **Surface "lawn" and few close to surface** |
|  | **16** | **20** | **0** | **51-75** | **9** | **Y** | **N** | **Moderate** | **Moderate** | **Surface "lawn" and few close to surface** |
|  | **17** | **20** | **1** | **26-50** | **9** | **Y** | **N** | **Moderate** | **Mild** | **Surface "lawn" and few close to surface** |
|  | **18** | **19** | **1** | **51-75** | **10** | **Y** | **N** | **Moderate** | **Moderate** | **Surface "lawn" and few close to surface** |
|  | 19 | 24 | 5 | 26-50 | 8 | N | N | Mild | Rare | Few close to surface |
|  | **20** | **25** | **2** | **51-75** | **10** | **Y** | **N** | **Moderate** | **Moderate** | **Surface "lawn" and few close to surface** |
|  | 21 | 22 | 0 | 0-25 | 4 | N | N | Mild | Mild | Few close to surface |
|  | **22** | **19** | **1** | **26-50** | **9** | **Y** | **N** | **Mild** | **Mild** | **Few close to surface** |
|  | **23** | **27** | **1** | **51-75** | **13** | **Y** | **N** | **Moderate** | **Mild** | **Surface "lawn" and few close to surface** |
|  | **24** | **21** | **1** | **51-75** | **10** | **Y** | **N** | **Moderate** | **Mild** | **Few close to surface** |

^a^ 0-25%, 26-50%, 51-75%, 76-99% and 100% field of view at 100x magnification

^b^ at 400x high powered field (HPF) and taken at section where glands were straightest

^c^ Y, present; N, absent

^d^ WNL (no epithelial cell injury), rare (attenuation, necrosis, loss or degeneration in a single focal section), mild (attenuation, necrosis, loss or degeneration of focal to multifocal areas comprising <5% of surface mucosal area), moderate (attenuation, necrosis, loss or degeneration of focal to multifocal areas comprising 5-15% of surface mucosal area), marked (attenuation, necrosis, loss or degeneration of multifocal to diffuse areas comprising >15% of surface mucosal area)
