# Supplementary material for: An atypical weakly haemolytic strain of Brachyspira hyodysenteriae is avirulent and can be used to protect pigs from developing swine dysentery
Source: Vet Res. 2019 Jun 19;50:47. doi: 10.1186/s13567-019-0668-5 (PMC6585146; doi:10.1186/s13567-019-0668-5)
Supplement: Supplementary file 2 — Additional file 2. Changes in cell numbers observed at pm in the large intestine of pigs in experiment 3. Pigs marked in bold had gross pathological changes observed at pm. [file 13567_2019_668_MOESM2_ESM.docx]

**Additional file 2 Table showing changes in cell numbers observed at pm in the large intestine of pigs in experiment 3.** Pigs marked in bold had gross pathological changes observed at pm.

| Pig Group | Pig Number | Intra-epithelial lymphocytes^a^ | Lamina Propria | | |
| --- | --- | --- | --- | --- | --- |
|  |  |  | Lymphocytes and plasma cells^b^ | Macrophages^c^ | Neutrophils^d^ |
| A | **1** | **WNL** | **WNL** | **WNL** | **WNL** |
|  | 2 | WNL | WNL | WNL | WNL |
|  | 3 | WNL | WNL | WNL | WNL |
|  | 4 | WNL | Mild | WNL | WNL |
|  | 5 | WNL | WNL | WNL | WNL |
|  | **6** | **WNL** | **WNL** | **WNL** | **WNL** |
|  | 7 | WNL | WNL | WNL | WNL |
|  | 8 | WNL | WNL | WNL | WNL |
|  | 9 | WNL | WNL | WNL | WNL |
|  | **10** | **WNL** | **WNL** | **WNL** | **WNL** |
|  | 11 | WNL | WNL | WNL | WNL |
|  | 12 | WNL | WNL | WNL | WNL |
| B | **13** | **WNL** | **WNL** | **Mild** | **WNL** |
|  | **14** | **WNL** | **Mild** | **Mild** | **Moderate** |
|  | **15** | **WNL** | **Mild** | **Mild** | **Moderate** |
|  | **16** | **WNL** | **Mild** | **WNL** | **Moderate** |
|  | **17** | **WNL** | **Mild** | **WNL** | **Moderate** |
|  | **18** | **WNL** | **Mild** | **WNL** | **Moderate** |
|  | 19 | WNL | WNL | WNL | Mild |
|  | **20** | **WNL** | **Mild** | **Mild** | **Moderate** |
|  | 21 | WNL | Mild | WNL | Mild |
|  | **22** | **WNL** | **Mild** | **WNL** | **Mild** |
|  | **23** | **WNL** | **Mild** | **Mild** | **Moderate** |
|  | **24** | **WNL** | **Mild** | **WNL** | **Moderate** |

^a^ WNL (no epithelial cell injury), rare (attenuation, necrosis, loss or degeneration in a single focal section), mild (attenuation, necrosis, loss or degeneration of focal to multifocal areas comprising <5% of surface mucosal area), moderate (attenuation, necrosis, loss or degeneration of focal to multifocal areas comprising 5-15% of surface mucosal area), marked (attenuation, necrosis, loss or degeneration of multifocal to diffuse areas comprising >15% of surface mucosal area).

^b^ WNL (5-15 per x400 HPF stretch), mild increase (15-30 per x400 HPF stretch), moderate increase (30-50 per x400 HPF stretch and may be clustered), marked increase (50-100 per x400 HPF stretch and may be clustered).

^c^ WNL (crypts separated by up to 5 cells), mild (lymphocytes and plasma cells may fill inter-cryptal region but do not disrupt normal perpendicular architecture), moderate (lymphocytes and plasma cells fill inter-cryptal region and moderately separate crypts), marked (lymphocytes and plasma cells diffusely distributed throughout the lamina propria and disrupt, distort or obliterate cryptal micro-architecture).

^d^ WNL (occasional scattered macrophages), mild (up to 20 cells per x400 HPF and occasionally forming clusters of up to 5 cells), moderate (up to 40 cells per 400x HPF and frequently forming small aggregates), marked (macrophages are the dominant population forming a diffuse sheet of cells throughout the lamina propria).
